# Association between changes in self-esteem and smartphone dependency among Korean adolescents

**DOI:** 10.1371/journal.pone.0338094

**Published:** 2025-12-10

**Authors:** Ah Jung Ko, Yu Shin Park, Jinhyun Kim, Eun-Cheol Park

**Affiliations:** 1 Department of Health Policy & Management, Graduate School of Public Health, Yonsei University, Seoul, Republic of Korea; 2 Institute of Health Services Research, Yonsei University, Seoul, Republic of Korea; 3 Department of Public Health, Graduate School, Yonsei University, Seoul, Korea; 4 Department of Preventive Medicine, Yonsei University College of Medicine, Seoul, Republic of Korea; 5 Department of Psychiatry, Yonsei University Hospital, Seoul, Republic of Korea; Southwest University, CHINA

## Abstract

Increasing smartphone ownership across all age groups has raised concerns about the rising risk of smartphone addiction, particularly during adolescence when self-esteem development is critical. This study aimed to investigate the association between changes in self-esteem and smartphone dependency over a five-year period using data from the Korean Children and Youth Panel Survey (2018–2022). A total of 1,971 participants from the middle school 1st grade cohort were analyzed. Self-esteem was measured annually using the Rosenberg Self-Esteem Scale, with a cutoff of 25 points classifying individuals into high and low self-esteem groups. Self-esteem was measured annually, and year-to-year change variables were created by comparing each participant’s score to that of the previous year. These lagged changes were used to assess their association with smartphone dependency measured at the same time point: (1) good → good, (2) good → poor, (3) poor → good, and (4) poor → poor. Smartphone dependency was assessed using the Smartphone Addiction Proneness Scale, with measurements taken at each annual survey point. The Generalized Estimating Equations model was used to analyze the association between self-esteem changes and smartphone dependency. The results indicated that both males and females with consistently low self-esteem or those whose self-esteem declined were more likely to exhibit smartphone dependency compared to those with consistently high self-esteem (females: good → poor: adjusted odds ratio (OR) 2.19, 95% confidence interval (CI) 1.78–2.71; poor → poor: adjusted OR 2.21, 95% CI 1.81–2.70; males: good → poor: adjusted OR 2.09, 95% CI 1.76–2.50; poor → poor: adjusted OR 2.04, 95% CI 1.70–2.48). These findings emphasize that stable self-esteem during adolescence may reduce the risk of smartphone dependency and related health issues.

## Introduction

Since the emergence of smartphones, their usage has steadily increased, with the age of users decreasing over time [1,2]. Smartphone ownership now exceeds half of the world’s population, with highest ownership rates among adolescents and young adults [2]. In Asian countries, the mobile phone penetration rate among individuals aged 12 and above is approximately 80% [3]. Smartphones provide various functions, such as internet access and communication, allowing users to accomplish a wide range of tasks. Moreover, they are convenient to carry [4,5]. These characteristics of smartphones are both advantageous and disadvantageous [6]. Some individuals may become addicted to smartphones to the extent that they interfere with their daily lives, surpassing excessive use [7,8] As ownership rates rise, so does the risk of smartphone addiction [9]. This can lead to further negative consequences, especially for adolescents with underdeveloped self-control abilities [10,11]. Consequently, ensuring proper smartphone use among adolescents has become a significant challenge, with heightened social concerns regarding the negative impacts of smartphone dependency on this demographic [9,12]. Previous research has indicated that excessive smartphone use can lead to attention deficits, depression, anxiety, and decreased sleep quality [13–15], as well as increased likelihood of adolescents engaging in problematic behaviors [16].

Self-esteem refers to how one values oneself [17]. It varies depending on the sex and age of the individual [18]. Because self-esteem is a subjective evaluation, it does not necessarily reflect objective factors [19]. Adolescence is a period characterized by rapid physiological changes, such as increased secretion of certain hormones, along with significant fluctuations in physical and psychological aspects [20,21]. Consequently, self-esteem undergoes changes during this period [19]. Furthermore, adolescence is a period of self-identity establishment, during which high self-esteem fosters a positive self-perception [22], while low self-esteem not only makes individuals sensitive to negative feedback and less responsive to positive feedback [23], but also increases aggressiveness and leads to a decline in everyday abilities [24]. Emotionally, this can trigger depression, while behaviorally, it can lead to antisocial behavior [25].

Adolescents with low self-esteem tend to internalize their anxiety, leading them to find greater enjoyment in interacting with others online rather than offline through the “false self” presented in the anonymous space of the internet [26,27]. According to Rosenberg’s self-esteem theory, individuals with low self-esteem may create a ‘false self’ to gain external validation and compensate for their perceived inadequacies [27,28]. According to psychological theories of identity development—such as Erikson’s psychosocial theory—adolescence is a critical period in which personal identity is still forming. During this stage, individuals may construct an ‘idealized self’ in online environments to explore different facets of their identity and seek social validation [28,29]. The anonymity and curated nature of digital interactions provide a space where they can present a more idealized version of themselves, often differing significantly from their real self [29]. Furthermore, adolescents who are more likely to develop smartphone addiction are often characterized by higher levels of anxiety and lower self-esteem compared to their peers [28].

While previous studies have shown that low self-esteem is associated with increased smartphone dependency, most have used cross-sectional designs, limiting insights into how self-esteem changes over time affect smartphone use [28]. Adolescents do not maintain a fixed level of self-esteem during this developmental stage rather, their self-worth often fluctuates in response to social, academic, and psychological factors [18,19]. These fluctuations may be more predictive of maladaptive behaviors, such as excessive smartphone use, than self-esteem measured at a single point in time [14,28]. For instance, a decline in self-esteem may trigger compensatory behaviors like increased online engagement, while an improvement in self-esteem may reduce reliance on smartphones for emotional support [28]. Therefore, examining the dynamic trajectories of self-esteem may provide deeper insights into the mechanisms linking adolescent development with problematic smartphone use [30]. This perspective is supported by prior research emphasizing the developmental significance and predictive utility of changes in self-esteem during adolescence, rather than static measurements [30,31]. We hypothesize that adolescents with declining self-esteem (good → poor) or consistently low self-esteem (poor → poor) are at greater risk of smartphone dependency. This study addresses this gap by examining the year-to-year (lagged) relationship between changes in self-esteem and smartphone dependency using longitudinal panel data.

## Methods

### Data

This study utilized data from the Korea Children and Youth Panel Survey (KCYPS), conducted from 2018 to 2022. This is an annual longitudinal study conducted by the National Youth Policy Institute [32] to examine various aspects ranging from the growth of children and adolescents to psychological issues and living environments [33]. The survey employed a multi-stage stratified cluster sampling method, with schools as the primary units [34] and targeting students aged 15 in 2018 [13]. The questionnaire included inquiries about academic achievement, peer relationships, parental relationships, and emotional issues, among others [13].

### Study population

In 2018, a total of 2,590 students in their first year of middle school were recruited as the baseline cohort of the Korean Children and Youth Panel Survey (KCYPS). These participants were followed annually over a five-year period (2018–2022), with a panel retention rate of 85.9%. Attrition was primarily due to school transfers or voluntary withdrawal from the study. Participants who did not own a mobile phone at the baseline year, as well as those with missing responses in the independent variable (self-esteem change) or dependent variable (smartphone dependency), were excluded from the final analysis. As a result, a total of 1,971 participants (1,041 males, 930 females) were included in the study. Data collection was conducted through individual interviews using tablet PCs (TAPI method), encompassing a wide range of variables including lifestyle, cognitive development, mental and physical health, and environmental factors such as family, school, and peer relationships, ensuring consistent methodology throughout the study period.

### Variables

The dependent variable was smartphone dependency, measured annually from 2018 to 2022 using the Smartphone Addiction Proneness Scale [35], comprising 15 items rated on a 4-point Likert scale. The total score ranges from 15 to 60, with three items reverse-scored [35]. The scale consists of four factors: Factor 1 (disturbance of adaptive functions), Factor 2 (virtual life orientation), Factor 3 (withdrawal), and Factor 4 (tolerance) [16]. Participants were categorized into normal (41 points or below), moderate (42–44 points, Factor 1 score of 14 or higher, Factor 3 score of 12 or higher, or Factor 4 score of 13 or higher), and severe (45 points or higher or Factor 1 score of 16 or higher, Factor 3 score of 13 or higher, or Factor 4 score of 14 or higher) [13].

The independent variable was self-esteem, measured using the Korean version of the Rosenberg Self-Esteem Scale [36], comprising 10 items rated on a 4-point Likert scale [27]. Among the 10 items, 5 were reverse scored [37]. The total score ranges from 10 to 40 points [37]. Self-esteem was categorized as low if it was 25 points or below, and as normal or higher if it exceeded 25 points [37]. The primary variable of interest, self-esteem change, was categorized into four groups: [1] good → good, [2] good → poor, [3] poor → good, [4] poor → poor. Self-esteem change was calculated as a year-to-year lagged variable, comparing each year’s score to the previous year’s score for the same participant. Therefore, participants contributed multiple observations (i.e., four transitions from 2018 → 2019, 2019 → 2020, 2020 → 2021, and 2021 → 2022), and these changes were linked to smartphone dependency measured at each corresponding year. Participants were not classified into a single fixed trajectory group; rather, each annual transition was treated as an independent time point within the GEE model framework.

The covariates included sociodemographic factors such as gender, family type (Two parents, other guardian), city (Capital, Non-Capital), household income (High, Middle, Low), and health-related variables including smoking status (Smoker, Non-smoker), alcohol consumption (Drinker, Non-drinker), body mass index (Underweight, Normal, Overweight), physical activity (Yes, No), subjective health status (High, Middle, Low), quality of sleep (Good, Poor), life satisfaction (Good, Poor), and self-esteem calculated in the previous year. Household income was categorized into Low (no income to 3 million KRW), Middle (3–6 million KRW), and High (6 million KRW and above). Smoking and alcohol consumption were assessed using frequency questions, with participants classified as smokers or drinkers if they reported any use beyond “Never.” Physical activity was evaluated based on weekly exercise duration, defining participants as active if they engaged in 1 hour or more of physical activity. Sleep quality was measured on a 4-point Likert scale and categorized into Good and Poor. All covariates, including sociodemographic factors and health-related variables, were assessed using self-reported questionnaires. Previous studies have shown that family structure, socioeconomic status, residential areas, and health behaviors influence adolescent self-esteem [38].

### Statistical analysis

Chi-square tests were conducted to examine the general characteristics of the study participants. Subsequently, for the regression analysis of self-esteem changes in relation to smartphone dependency and covariates, a Generalized Estimating Equations (GEE) model including a logit link was employed. GEE was used to account for the correlated structure of repeated observations within individuals across five annual survey waves. The main analytical goal was to estimate population-averaged effects of lagged self-esteem change variables on the likelihood of reporting smartphone dependency over time. The time variable was waves, (i.e., every year), and the main outcomes were expressed as odds ratios (ORs) with 95% confidence intervals (CIs). All analyses were performed using SAS version 9.4, and significance was considered at p < 0.05.

## Results

Table 1 compares the demographic characteristics of the study population. Smartphone dependency was observed among 499 (47.9%) males and 444 (47.7%) females. A statistically significant difference in smartphone dependency was also observed according to changes in self-esteem.

**Table 1:** Baseline characteristics of the study population (2018 → 2019) according to smartphone dependency.

Variables		Smartphone Dependency									
		Male				p-value	Female				p-value
		Normal		Dependence			Normal		Dependence		
		N	(%)	N	(%)		N	(%)	N	(%)	
**Total(N = 1,971)**		542	(52.1)	499	(47.9)		486	(52.3)	444	(47.7)	
**Self-Esteem**						<.0001					<.0001
	Good - > Good	309	(59.7)	209	(40.3)		228	(68.5)	105	(31.5)	
	Good - > Poor	88	(41.3)	125	(58.7)		76	(44.7)	94	(55.3)	
	Poor - > Good	77	(56.2)	60	(43.8)		95	(59.4)	65	(40.6)	
	Poor - > Poor	68	(39.3)	105	(60.7)		87	(32.6)	180	(67.4)	
**Family type**						0.5460					0.1475
	Two parents	486	(51.8)	453	(48.2)		433	(51.5)	408	(48.5)	
	Other guardian	56	(54.9)	46	(45.1)		53	(59.6)	36	(40.4)	
**City**						0.4546					0.7674
	Capital	266	(51.4)	252	(48.6)		251	(52.7)	225	(47.3)	
	Non-Capital	276	(52.8)	247	(47.2)		235	(51.8)	219	(48.2)	
**Household income**						0.1959					<.0001
	High	172	(56.4)	133	(43.6)		152	(65.0)	82	(35.0)	
	Middle	305	(50.1)	304	(49.9)		274	(48.2)	294	(51.8)	
	Low	65	(51.6)	61	(48.4)		60	(46.9)	68	(53.1)	
**Smoking status**						<.0001					0.0004
	No	534	(53.7)	461	(46.3)		482	(53.2)	424	(46.8)	
	Yes	8	(17.4)	38	(82.6)		4	(16.7)	20	(83.3)	
**Alcohol status**						0.0015					0.1126
	No	521	(53.3)	456	(46.7)		470	(52.8)	420	(47.2)	
	Yes	21	(32.8)	43	(67.2)		16	(40.0)	24	(60.0)	
**BMI**						0.6497					0.8427
	Underweight	128	(49.8)	129	(50.2)		156	(52.3)	142	(47.7)	
	Normal	238	(52.2)	218	(47.8)		262	(52.8)	234	(47.2)	
	Overweight	176	(53.7)	152	(46.3)		68	(50.0)	68	(50.0)	
**Physical activity**						0.1011					0.3464
	Yes	486	(53.0)	431	(47.0)		323	(53.4)	282	(46.6)	
	No	56	(45.2)	68	(54.8)		163	(50.2)	162	(49.8)	
**Self-reported health status**						0.0042					0.0037
	High	204	(59.1)	141	(40.9)		152	(60.8)	98	(39.2)	
	Middle	302	(49.1)	313	(50.9)		297	(49.9)	298	(50.1)	
	Low	36	(44.4)	45	(55.6)		37	(43.5)	48	(56.5)	
**Sleep quality**						0.0543					<.0001
	Good	487	(53.2)	429	(46.8)		448	(55.2)	364	(44.8)	
	Poor	55	(44.0)	70	(56.0)		38	(32.2)	80	(67.8)	
**Satisfaction with life**						<.0001					<.0001
	Good	246	(63.2)	143	(36.8)		188	(64.6)	103	(35.4)	
	Poor	296	(45.4)	356	(54.6)		298	(46.6)	341	(53.4)	
**Dependence for the previous year**						0.3135					0.0009
	Normal	296	(50.7)	288	(49.3)		289	(57.2)	216	(42.8)	
	Dependency	246	(53.8)	211	(46.2)		197	(46.4)	228	(53.6)	

Table 2 presents the results of GEE analysis adjusting for covariates to examine the association between changes in self-esteem and smartphone dependency. Females with changes in reported self-esteem from good to poor and who remained with poor self-esteem across modeled time points (good - > poor: adjusted OR 2.19, 95% CI 1.78–2.71; poor - > poor: adjusted OR 2.21, 95% CI 1.81–2.70). Similarly, males with poor self-esteem were significantly more likely to exhibit smartphone dependency than were those with good self-esteem (good - > poor: adjusted OR 2.09, 95% CI 1.76–2.50; poor - > poor: adjusted OR 2.04, 95% CI 1.70–2.48).

**Table 2:** Results of GEE analysis of factors associated with smartphone dependency in 2018 to 2022.

Variables		Smartphone Dependency			
		Male		Female	
		OR	95%CI	OR	95%CI
**Self-Esteem**					
	Good - > Good	1.00		1.00	
	Good - > Poor	2.09	(1.76–2.50)	2.19	(1.78–2.71)
	Poor - > Good	1.21	(1.01–1.46)	1.04	(0.85–1.28)
	Poor - > Poor	2.04	(1.70–2.48)	2.21	(1.81–2.70)
**Family type**					
	Two parents	1.00		1.00	
	Other guardian	0.85	(0.66–1.12)	0.77	(0.59–1.03)
**City**					
	Capital	1.00		1.00	
	Non-Capital	1.02	(0.88–1.19)	1.02	(0.87–1.19)
**Household income**					
	High	1.00		1.00	
	Middle	1.23	(1.06–1.45)	1.37	(1.17–1.61)
	Low	1.26	(0.96–1.66)	1.27	(0.97–1.67)
**Smoking status**					
	No	1.00		1.00	
	Yes	1.58	(1.09–2.31)	2.37	(1.07–5.26)
**Alcohol status**					
	No	1.00		1.00	
	Yes	1.18	(0.94–1.51)	2.06	(1.27–3.37)
**BMI**					
	Underweight	1.03	(0.86–1.25)	0.96	(0.81–1.16)
	Normal	1.00		1.00	
	Overweight	0.88	(0.76–1.03)	0.99	(0.80–1.24)
**Physical activity**					
	Yes	1.00		1.00	
	No	0.94	(0.80–1.11)	1.08	(0.94–1.25)
**Self-reported health status**					
	High	1.00		1.00	
	Middle	1.25	(1.08–1.45)	1.15	(0.98–1.36)
	Low	1.14	(0.87–1.52)	1.24	(0.93–1.66)
**Sleep quality**					
	Good	1.00		1.00	
	Poor	1.16	(0.96–1.42)	1.35	(1.09–1.69)
**Satisfaction with life**					
	Good	1.00		1.00	
	Poor	1.62	(1.40–1.89)	1.29	(1.09–1.54)
**Dependence for the previous year**					
	Normal	1.00		1.00	
	Dependency	1.33	(1.17–1.52)	1.95	(1.70–2.26)

Abbreviations: GEE, Generalized estimating equation.

Table 3 presents the subgroup analysis of the relationship between self-esteem and smartphone dependency, stratified by independent variables. Among obese female students, the odds of being dependent on mobile phones were 0.96 (adjusted OR 0.96, 95% CI 0.54–1.72) when self-esteem improved, while it was 1.54 (adjusted OR 1.54, 95% CI 0.96–2.49). when self-esteem remained consistently low. When self-esteem changed from good to poor, the odds of being dependent on mobile phones were the highest at 2.04 (adjusted OR 1.54, 95% CI 1.21–3.44). A similar trend was observed in male students, with odds of 2.21 (adjusted OR 2.21, 95% CI 1.64–2.98) for mobile phone dependence when self-esteem changed from good to poor, and 2.00 (adjusted OR 2.00, 95% CI 1.47–2.73) when self-esteem remained consistently low.

**Table 3:** Subgroup analysis of the relationship between self-esteem and smartphone dependency, stratified by independent variables in 2018 to 2022.

Variables		Smartphone Dependency													
		Male							Female						
		Good -> Good	Good -> Poor		Poor -> Good		Poor -> Poor		Good -> Good	Good -> Poor		Poor -> Good		Poor -> Poor	
			OR	95%CI	OR	95%CI	OR	95%CI		OR	95%CI	OR	95%CI	OR	95%CI
**City**	** **	** **	** **	** **	** **	** **	** **	** **	** **	** **	** **	** **	** **	** **	** **
** **	**Capital**	**1.00**	**2.15**	**(1.69–2.76)**	**1.41**	**(1.09-1.83)**	**2.18**	**(1.64-2.90)**	**1.00**	**2.92**	**(2.20-3.90)**	**1.10**	**(0.83-1.45)**	**2.69**	**(2.07-3.53)**
** **	**Non-Capital**	**1.00**	**2.02**	**(1.57–2.62)**	**1.17**	**(0.90-1.54)**	**2.22**	**(1.68-2.95)**	**1.00**	**1.64**	**(1.20-2.25)**	**1.11**	**(0.84-1.48)**	**2.06**	**(1.52-2.81)**
** **	**Rural**	**1.00**	**2.37**	**(1.65-3.42)**	**1.36**	**(0.90-2.08)**	**3.14**	**(2.00-4.94)**	**1.00**	**0.00**	**(0.00-0.00)**	**0.00**	**(0.00-0.00)**	**0.00**	**(0.00-0.00)**
**Household income**	** **	** **	** **	** **	** **	** **	** **	** **	** **	** **	** **	** **	** **	** **	** **
** **	**High**	**1.00**	**2.20**	**(1.62–3.01)**	**1.39**	**(1.00-1.93)**	**2.58**	**(1.86-3.60)**	**1.00**	**2.66**	**(1.82-3.89)**	**1.11**	**(0.78-1.60)**	**2.35**	**(1.63-3.40)**
** **	**Middle**	**1.00**	**2.15**	**(1.72–2.71)**	**1.18**	**(0.92-1.51)**	**2.10**	**(1.63-2.71)**	**1.00**	**1.99**	**(1.51-2.63)**	**0.96**	**(0.72-1.28)**	**2.01**	**(1.57-2.59)**
** **	**Low**	**1.00**	**1.77**	**(0.96–3.27)**	**1.40**	**(0.75-2.66)**	**1.67**	**(0.98-2.87)**	**1.00**	**2.08**	**(0.98-4.41)**	**1.11**	**(0.57-2.18)**	**2.85**	**(1.49-5.44)**
**BMI**	** **	** **	** **	** **	** **	** **	** **	** **	** **	** **	** **	** **	** **	** **	** **
** **	**Underweight**	**1.00**	**2.32**	**(1.45–3.71)**	**0.85**	**(0.53-1.38)**	**2.18**	**(1.27-3.76)**	**1.00**	**1.65**	**(1.10-2.48)**	**1.17**	**(0.78-1.75)**	**2.70**	**(1.84-3.96)**
** **	**Normal**	**1.00**	**2.18**	**(1.69–2.81)**	**1.32**	**(1.02-1.73)**	**2.22**	**(1.70-2.90)**	**1.00**	**2.50**	**(1.89-3.30)**	**1.00**	**(0.77-1.31)**	**2.27**	**(1.75-2.95)**
** **	**Overweight**	**1.00**	**2.21**	**(1.64–2.98)**	**1.19**	**(0.87-1.64)**	**2.00**	**(1.47-2.73)**	**1.00**	**2.04**	**(1.21-3.44)**	**0.96**	**(0.54-1.72)**	**1.54**	**(0.96-2.49)**
**Physical activity**	** **	** **	** **	** **	** **	** **	** **	** **	** **	** **	** **	** **	** **	** **	** **
** **	**Yes**	**1.00**	**2.34**	**(1.91–2.86)**	**1.20**	**(0.98-1.49)**	**2.20**	**(1.77-2.74)**	**1.00**	**2.38**	**(1.77-3.21)**	**0.85**	**(0.64-1.14)**	**2.42**	**(1.85-3.17)**
** **	**No**	**1.00**	**1.61**	**(1.10–2.36)**	**1.14**	**(0.75-1.77)**	**1.76**	**(1.19-2.60)**	**1.00**	**1.98**	**(1.47-2.68)**	**1.28**	**(0.97-1.71)**	**2.08**	**(1.57-2.76)**
**Self-reported health status**	** **	** **	** **	** **	** **	** **	** **	** **	** **	** **	** **	** **	** **	** **	** **
** **	**High**	**1.00**	**2.49**	**(1.81–3.44)**	**1.42**	**(1.04-1.95)**	**2.16**	**(1.51-3.09)**	**1.00**	**1.68**	**(1.14-2.50)**	**1.01**	**(0.71-1.46)**	**1.28**	**(0.88-1.86)**
** **	**Middle**	**1.00**	**1.89**	**(1.52–2.38)**	**1.09**	**(0.86-1.40)**	**1.97**	**(1.56-2.51)**	**1.00**	**2.60**	**(2.01-3.39)**	**1.12**	**(0.87-1.46)**	**3.01**	**(2.37-3.84)**
** **	**Low**	**1.00**	**2.30**	**(1.33–4.00)**	**1.04**	**(0.54-2.03)**	**1.93**	**(1.10-3.41)**	**1.00**	**1.15**	**(0.51-2.61)**	**0.90**	**(0.33-2.46)**	**1.00**	**(0.48-2.11)**
**Satisfaction with life**	** **	** **	** **	** **	** **	** **	** **	** **	** **	** **	** **	** **	** **	** **	** **
** **	**Good**	**1.00**	**2.12**	**(1.51–2.98)**	**0.93**	**(0.66-1.31)**	**2.21**	**(1.49-3.29)**	**1.00**	**2.43**	**(1.55-3.84)**	**1.36**	**(0.91-2.05)**	**3.61**	**(2.29-5.70)**
** **	**Poor**	**1.00**	**2.10**	**(1.72–2.59)**	**1.33**	**(1.34-1.07)**	**2.01**	**(1.64-2.49)**	**1.00**	**2.09**	**(1.65-2.66)**	**0.91**	**(0.72-1.17)**	**1.97**	**(1.58-2.46)**

Table 4 presents the results of subgroup analysis by the dependent variable—smartphone dependency—which was classified as moderate or severe. Compared with individuals who exhibited no smartphone dependency and consistently good self-esteem, the odds of developing severe smartphone dependency increased when self-esteem changed from good to poor (females: adjusted OR 2.14, 95% CI 1.78–2.58, males: adjusted OR 2.20, 95% CI 1.76–2.75) and when it remained consistently poor (females: adjusted OR 1.92, 95% CI 1.59–2.34, males: adjusted OR 2.10, 95% CI 1.72–2.58). In contrast, changes from poor to good self-esteem were not associated with statistically significant differences in the odds of reporting smartphone dependency (adjusted OR 1.09, 95% CI 0.90–1.34), and 0.99 for females (adjusted OR 0.99, 95% CI 0.80–1.24).

**Table 4:** Results of subgroup analysis stratified by dependent variables in 2018 to 2022.

Variables		Smartphone Dependency						
		Not a thing			Moderate		Severe	
		OR	95%CI		OR	95%CI	OR	95%CI
**Male**								
**Self-Esteem**								
	Good -> Good	1.00						
	Good -> Poor				3.36	(1.93-5.86)	2.14	(1.78-2.58)
	Poor -> Good				2.47	(1.40-4.36)	1.09	(0.90-1.34)
	Poor -> Poor				3.69	(2.23-6.14)	1.92	(1.59-2.34)
**Female**								
**Self-Esteem**								
	Good -> Good	1.00						
	Good -> Poor				2.38	(1.34-4.25)	2.20	(1.76-2.75)
	Poor -> Good				0.91	(0.50-1.68)	0.99	(0.80-1.24)
	Poor -> Poor				2.72	(1.55-4.77)	2.10	(1.72-2.58)

## Discussion

The study explored the relationship between changes in self-esteem and smartphone dependency using longitudinal panel survey data from KCYPS. The groups of individuals with poor self-esteem exhibited a higher likelihood of being dependent on smartphones than did the group with good self-esteem. Males whose self-esteem changed from good to poor exhibited the highest odds of smartphone dependency. Additionally, all groups with poor self-esteem were significantly associated with moderate and severe smartphone dependency. The group with the highest odds of developing severe smartphone dependency in both males and females was the one in which self-esteem changed from good to poor.

Previous research suggests that low self-esteem is associated with addiction [39,40]. Adolescents with low self-esteem exhibited higher prevalence rates of internet and social media addiction than those with high self-esteem [39,40]. This aligns with the findings of this study, which revealed that groups with low self-esteem had higher levels of smartphone dependency. The relationship between self-esteem and internet usage was more pronounced among male students, while the relationship between self-esteem and social media usage was more pronounced among female students [39,40]. The analysis of self-esteem and smartphone dependency in this study was significant for both male and female students, although the results were more pronounced among female students.

In addition to the primary findings, several subgroup patterns warrant further discussion. Notably, gender differences were observed in the association between changes in self-esteem and smartphone dependency. While both males and females with declining or persistently low self-esteem exhibited increased odds of smartphone dependency, the effect was more pronounced in females. This finding aligns with prior research suggesting that adolescent girls may be more emotionally vulnerable to fluctuations in self-worth, which can amplify reliance on digital devices for social validation or emotional regulation [18,40]. From a clinical and policy perspective, this underscores the importance of gender-sensitive approaches to prevention and intervention programs aimed at reducing problematic smartphone use, especially among adolescent girls undergoing self-esteem instability.

Furthermore, our analysis also identified that overweight or obese adolescents were more likely to report both lower self-esteem and higher smartphone dependency. Body image can contribute to self-esteem, which in turn may be linked to smartphone dependency, forming a potential triadic relationship [21,28]. Previous studies have shown that a high BMI can influence the development of negative self-concept and stigmatization within peer groups [21,41]. Future studies should further investigate these interrelationships and consider the role of body image and physical health in the development of digital dependencies among youth.

The mutual impacts of low self-esteem and smartphone dependency have been extensively studied [28,42]. This study presents the novel perspective that self-esteem influences smartphone dependency by analyzing the latter based on changes in the former.

Low self-esteem increases anxiety, which is associated with self-control [43], the ability to regulate and restrain oneself, considered a core function of the self [44]. Anxiety can diminish this self-control ability [44]. Individuals with weakened self-control may be more susceptible to addiction and may struggle to regulate their smartphone use, leading to increased dependency [28]. Consequently, low self-esteem can induce anxiety, which in turn decreases self-control and can increase smartphone dependency [28].

Adolescence is a period during which self-esteem can fluctuate due to interactions with various external events [38]. During this time, adolescents with low self-esteem may construct an “idealized self” in online environments, allowing them to engage in positive interactions with others [27,28]. Such experiences increase the risk of smartphone addiction among adolescents [29].

Individuals may immerse themselves in smartphone use as a means of avoiding negative emotions such as anxiety [43], as this offers them an escape from reality by alleviating the emotions encountered in daily life [43]. Smartphones are always accessible, providing an endless stream of stimulating content and enabling users to quickly become absorbed [45]. Thus, individuals may increase their smartphone usage to escape from negative emotions such as anxiety and depression, stemming from low self-esteem [28].

This study highlights the importance of focusing on year-to-year changes in self-esteem rather than treating it as a static trait [19]. By using a lagged approach, we examined how recent shifts in self-esteem were associated with smartphone dependency measured in the same year [31]. This design captures the temporal dynamics of adolescence, where self-worth may fluctuate in response to life events [30]. Such fluctuations may be more predictive of smartphone use than self-esteem measured at a single time point, as adolescents may turn to smartphones as a coping mechanism following a decline in self-esteem [28]. Our approach aligns with prior studies that emphasize the predictive value of self-esteem changes over time, rather than static measurements [19,31].

This study has several limitations. First, because the smartphone dependency scale was developed in Korea, its external validity may be limited. Second, the data relied on self-reporting by adolescents, which could lead to underestimation or overestimation of the variables included in the study. Despite these limitations, the study utilized nationally representative data, based on which it revealed the association between changes in smartphone dependency and self-esteem.

## 5. Conclusion

This study examined the relationship between changes in self-esteem and smartphone dependency during adolescence using longitudinal data. The findings suggest that a decline in self-esteem may lead to problematic smartphone use among adolescents, highlighting the influence of psychological fluctuations on digital behavior. Therefore, maintaining stable self-esteem may be a crucial factor in preventing problematic smartphone use. However, this study has the limitation of not being able to establish a clear causal relationship, indicating the need for future research utilizing experimental designs for further analysis.
